# Monoclonal Antibodies in Pregnancy of Patients with Systemic Lupus Erythematosus: Friend or Foe? A Case Report of a Patient with Multiple Pregnancies

**DOI:** 10.3390/antib15020032

**Published:** 2026-04-08

**Authors:** Chiara Orlandi, Angela Tincani, Micaela Fredi, Laura Andreoli, Francesca Crisafulli, Liala Moschetti, Cecilia Nalli, Maria Grazia Lazzaroni, Marco Taglietti, Matteo Filippini, Sonia Zatti, Laura Picciau, Franco Franceschini, Ilaria Cavazzana

**Affiliations:** 1 Rheumatology and Clinical Immunology, ASST Spedali Civili of Brescia, ERN-ReCONNET Center, 25123 Brescia, Italy; 2 Department of Clinical and Experimental Sciences, University of Brescia, 25123 Brescia, Italy; 3 Obstetrics and Gynecology, ASST Spedali Civili of Brescia, 25123 Brescia, Italy; 4 Neonatal Intensive Care Unit, Children’s Hospital, ASST Spedali Civili di Brescia, 25123 Brescia, Italy

**Keywords:** systemic lupus erythematosus, pregnancy, belimumab, lactation, fetal exposure, neonatal outcomes

## Abstract

Systemic lupus erythematosus (SLE) is an autoimmune disease that predominantly affects women of childbearing age, and active disease during pregnancy is associated with increased maternal and fetal morbidity. Belimumab is an effective biologic therapy for active SLE; however, its use during pregnancy has long been limited by the scarcity of safety data. Recent evidence and updated international recommendations suggest that belimumab may be considered in selected cases when required to maintain maternal disease control. We report the case of a woman with SLE who experienced three consecutive pregnancies with live births between 2019 and 2024 while receiving belimumab, allowing an intra-individual comparison of different exposure strategies. During the first pregnancy, belimumab was discontinued at conception and was followed by a disease flare in late pregnancy and postpartum. In the second and third pregnancies, belimumab was continued until gestational week 20 following shared decision-making with the patient; nevertheless, disease flares occurred during the third trimester of both pregnancies. All pregnancies resulted in live births at term, with no congenital anomalies, placental insufficiency, or fetal growth restriction. One neonate from the third pregnancy developed early-onset neonatal sepsis and meningitis, which resolved completely after antibiotic treatment. All children are currently growing and developing normally. This case supports a risk-adapted approach to belimumab use during pregnancy. In selected women with SLE at high risk of disease reactivation, continuation of belimumab until mid-gestation may contribute to improved maternal disease control without evident adverse fetal outcomes.

## 1. Introduction

SLE is an autoimmune disease that predominantly affects women of childbearing age. Pregnancy in patients with SLE may be complicated by disease flares and adverse pregnancy outcomes, including preeclampsia, fetal loss, preterm birth, fetal growth restriction, and hypertensive disorders of pregnancy [1,2]. Moreover, high disease activity during pregnancy and in the postpartum period may further contribute to unfavorable maternal and fetal outcomes, with increased morbidity and mortality [3]. For these reasons, adequate control of disease activity before, during, and after pregnancy is essential, considering that fetal health is closely linked to maternal well-being.

Currently, medications considered compatible with pregnancy include corticosteroids (CS), azathioprine (AZA), hydroxychloroquine (HCQ), cyclosporine (CYA), and tacrolimus (TAC). However, these therapies may be insufficient to achieve optimal disease control in some patients, particularly those with highly active SLE or lupus nephritis.

Belimumab (BEL) is a recombinant humanized IgG1λ monoclonal antibody directed against the soluble B-lymphocyte stimulator protein (BAFF/BLyS), thereby inhibiting B-cell survival. It was the first biologic agent approved for the treatment of active SLE and is currently indicated for the management of both renal and extrarenal SLE [4,5]. Despite its approval in 2011, belimumab has long not been recommended for use during pregnancy due to the limited availability of safety data.

In patients receiving belimumab, pregnancy planning requires careful evaluation of the risks and benefits of continuing or discontinuing treatment, with an individualized approach. Discontinuation of belimumab may be associated with an increased risk of disease flares, with potentially adverse consequences for both maternal and fetal outcomes. Conversely, the currently available evidence does not allow for a definitive assessment of the risks associated with continuing belimumab therapy during gestation.

In the 2024 update of the EULAR recommendations on the use of antirheumatic drugs during pregnancy, both belimumab and rituximab are included among the therapies that may be used, if necessary, to control maternal disease; however, for both agents, the available evidence remains limited [6]. In light of these considerations, further investigation into the safety profile of belimumab during pregnancy is warranted.

Therefore, we report the case of a woman who was treated with belimumab throughout three pregnancies and provide a summary of the available published data on the safety of belimumab during pregnancy.

## 2. Detailed Case Description

We report the case of a woman of African descent, born in 1987, with normal Body Mass Index and no history of smoking. Her medical history was notable for hypothyroidism and a secreting prolactinoma. She was diagnosed with SLE in 2010, at the age of 23, based on the presence of cutaneous manifestations, myositis, lymphadenopathies, Raynaud’s phenomenon, arthritis and leukopenia, together with supportive serological findings, including elevated anti-double-stranded DNA (anti-dsDNA) antibody titers, complement consumption, and positivity for anti-Ro/SSA and anti-U1RNP antibodies. Antiphospholipid antibodies were negative.

At diagnosis, she was treated with HCQ at a dose of 5 mg/kg/day, methotrexate (MTX) at 10 mg/week, and prednisone (PDN) at an initial dose of 40 mg/day and gradually tapered over two years to approximately 5 mg/day. One year later, due to persistent serological activity, MTX was discontinued and replaced with mycophenolate mofetil (MMF) at a dose of 2 g/day. In 2013, intravenous BEL at a dose of 10 mg/kg/month was added to the therapeutic regimen because of ongoing arthritis.

### 2.1. First Pregnancy

In 2017, the patient expressed a desire to conceive. At that time, her disease was moderately controlled with HCQ, PDN, MMF, and BEL. Despite persistent leukopenia and anti-dsDNA positivity, complement levels were within the normal range, and the SLE Disease Activity Index 2000 (SLEDAI-2K) score was 3. In the context of pregnancy planning, MMF was discontinued because of its known teratogenicity and replaced with CYA at a dose of 3 mg/kg/day, an immunosuppressive drug compatible with pregnancy. The remaining therapies were continued, as HCQ, low-dose PDN, and BEL because contributed to disease stability and were considered necessary to minimize the risk of disease flare prior to conception.

In March 2019, at the age of 32, the patient became pregnant. In accordance with the preconception plan and following shared decision-making process, BEL was discontinued at the time of pregnancy confirmation because of limited safety data during gestation. HCQ and low-dose PDN were continued, as well as vitamin D supplementation, and low-dose aspirin was initiated to reduce the risk of preeclampsia and placental-mediated complications. Folic acid was also started.

Due to anti-Ro/SSA antibody positivity, fetal cardiac surveillance with serial echocardiography was performed between gestational weeks 16 and 26 to screen for congenital atrioventricular block. The patient was followed monthly throughout the pregnancy. Fetal growth remained within normal limits, with no evidence of placental insufficiency.

**Figure 1:**
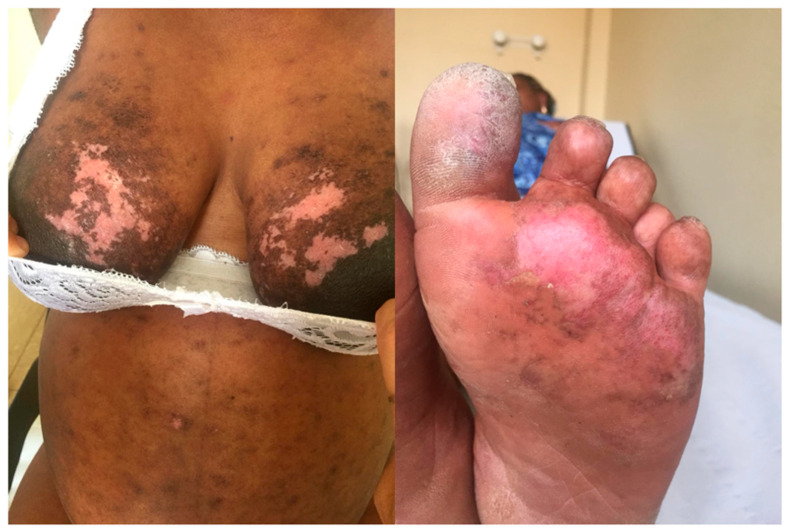
Cutaneous flare at 34 weeks of gestation during the patient’s first pregnancy, showing erythematous and depigmented lesions on the chest and foot.

At 34 weeks of gestation, the patient developed a cutaneous SLE flare (Figure 1), which was successfully treated with intravenous methylprednisolone (80 mg/day for three consecutive days). No obstetric complications were observed during the subsequent course of pregnancy. At 37 weeks of gestation, she underwent an induced vaginal delivery, resulting in the birth of a healthy female infant weighing 3020 g, with an Apgar score of 9 at 1 min. No congenital anomalies were detected at birth. The patient breastfed for approximately two months, by personal choice. Belimumab was restarted only after breastfeeding cessation, in accordance with the patient’s preference.

CYA was discontinued and both intravenous BEL at a dose of 10 mg/kg/month, and MMF at a dose of 2 g/day were reintroduced. At the time of MMF reinitiation, an intrauterine device (IUD) was placed for contraceptive purposes. In 2020, however, cutaneous disease activity worsened again. Before modifying systemic immunosuppressive therapy, alternative treatment options were evaluated. Dapsone was excluded because of the patient’s glucose-6-phosphate dehydrogenase deficiency, and thalidomide was not considered suitable owing to poor adherence to contraceptive measures. Topical tacrolimus therapy was also deemed inappropriate by the dermatologist because of the extensive cutaneous involvement.

**Figure 2:**
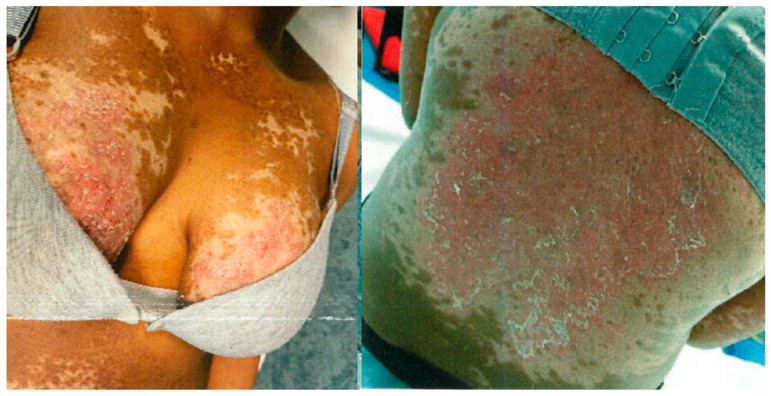
Cutaneous flare after pregnancy, showing erythematous and depigmented lesions on the chest and back.

Consequently, MMF was discontinued and MTX was reintroduced at a dose of 10 mg/week, together with high-dose PDN at 50 mg/day, subsequently tapered. Despite these therapeutic adjustments, disease control remained suboptimal throughout 2021 (Figure 2). Cyclosporine was therefore added and PDN was maintained at 25 mg/day, resulting in improved disease control and allowing a gradual reduction in the corticosteroid dose by approximately 50%.

### 2.2. Second Pregnancy

In 2022, the patient again expressed a desire to conceive. At that time, cutaneous and articular manifestations were well controlled. Persistent leukopenia was present, whereas anti-dsDNA antibodies and complement levels were within the normal range (SLEDAI-2K 1). Her ongoing therapy consisted of HCQ 5 mg/kg/day, PDN 12.5 mg/day, MTX 10 mg/week, CYA 3 mg/kg/day, and subcutaneous BEL 200 mg/week. In agreement with the patient, the therapeutic plan aimed to achieve stable disease control for at least eight months before methotrexate withdrawal, together with a gradual reduction in prednisone to approximately 5 mg/day, after which pregnancy planning was initiated.

In October 2022, the patient reported a positive pregnancy test, and low-dose aspirin and folic acid were initiated. In light of the significant disease flare that had occurred during the first pregnancy following its discontinuation, BEL was continued until gestational week 20. This decision was informed by both emerging evidence from the 2022 British Society for Rheumatology (BSR) guidelines, suggesting that continuation of belimumab during pregnancy may be considered in cases of severe disease activity when required to maintain maternal disease control, and emerging data from Belimumab pregnancy Registry and postmarketing reports [7,8,9].

The patient was closely monitored throughout pregnancy with regular clinical assessments and regular serial echocardiography for the congenital atrioventricular block screening. Fetal ultrasound examinations demonstrated normal fetal growth, with no evidence of placental insufficiency. At 33 weeks of gestation, she developed a mild cutaneous disease flare, which was initially managed by increasing the oral corticosteroid dosage at 10 mg/day. At 37 weeks of gestation, due to worsening cutaneous and articular manifestations, labor was induced. She underwent an induced vaginal delivery and delivered a healthy male infant weighing approximately 3000 g, without obstetric complications or congenital anomalies. The patient breastfed for approximately two months, according to the patient’s personal decision. In this case, belimumab therapy was promptly resumed in the postpartum period during breastfeeding because of the clinical need to better control disease activity, with subsequent clinical improvement. She also initiated a progestin-only oral contraceptive. At that time, only minimal cutaneous activity and mild leukopenia were noted (SLEDAI-2K 3). However, a few months later, in January 2024, the patient reported a third unplanned pregnancy.

### 2.3. Third Pregnancy

Management during the third pregnancy followed the same strategy as in the previous gestation, with BEL continued until gestational week 20 following shared decision-making. Fetal monitoring showed normal growth and no evidence of placental insufficiency. At approximately 29 weeks of gestation, the patient experienced a disease flare (cutaneous and articular), which significantly worsened by week 35, necessitating hospital admission and treatment with intravenous methylprednisolone pulses. Near term, the patient acquired SARS-CoV-2 infection, which had a mild course, not necessitating specific therapy; however, as a precautionary measure, the CYA dose was temporarily reduced to 100 mg/day. At 37 weeks of gestation, labor was induced, and she delivered a female infant weighing 2630 g. On the day of delivery and the following day, she received intravenous boluses of methylprednisolone at a dose of 40 mg. Shortly after birth, the newborn developed respiratory distress and was admitted to the neonatal intensive care unit. Sepsis and meningitis were diagnosed, with *Streptococcus agalactiae* identified as the causative agent. The infant responded well to antibiotic therapy and was discharged after two weeks, having made a full recovery. The patient did not breastfeed by personal choice.

**Figure 3:**
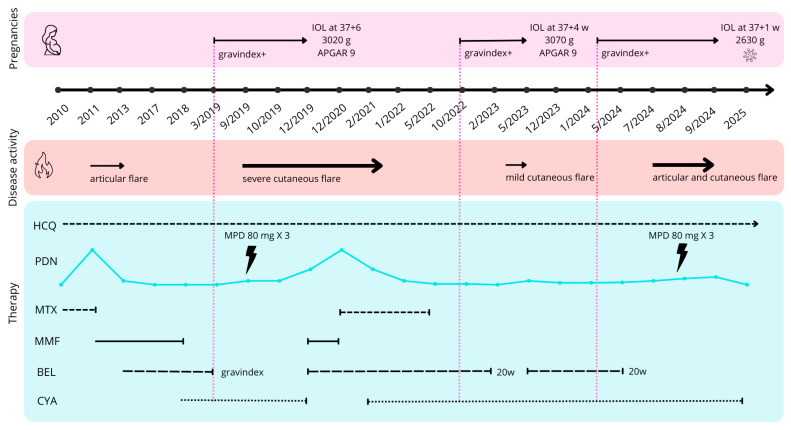
Timeline of pregnancies, disease activity, and treatments. The upper panel shows the patient’s three pregnancies, each marked by a positive pregnancy test (gravindex+) and ending with induction of labor (IOL) at 37 + 6, 37 + 4, and 37 + 1 weeks of gestation, respectively; neonatal birth weight and Apgar score are also reported. The middle panel depicts episodes of articular and cutaneous flares of different severity. The lower panel summarizes the longitudinal treatment course, including hydroxychloroquine (HCQ), prednisone (PDN), methotrexate (MTX), mycophenolate mofetil (MMF), belimumab (BEL), and cyclosporine A (CYA). The lightning bolt indicates high-dose intravenous methylprednisolone pulses, reported as MPD 80 mg × 3 (methylprednisolone 80 mg administered for three doses). Vertical pink dashed lines indicate the onset of each pregnancy, whereas arrows indicate the duration of pregnancies and flare episodes.

Pregnancy outcomes and treatment modifications are summarized in Figure 3.

### 2.4. Follow Up

In December 2024, treatment with anifrolumab was initiated. In January 2025, the patient opted for an IUD for long-term contraception. At the most recent follow-up, the disease was inactive, with no cutaneous manifestations and only mild leukopenia (SLEDAI-2K 1). The patient is currently receiving a low-dose corticosteroid regimen, consisting of prednisone 2.5 mg/day. Further tapering of prednisone is planned.

All three children are currently growing and developing normally. Aside from the youngest child’s neonatal infectious complication and a brief hospitalization of the first child for cow’s milk protein allergy, no major health issues or recurrent/severe infections have been reported during follow-up. Routine vaccinations were completed, with rotavirus vaccination postponed by six months in the second and third child compared with the standard schedule, which includes two doses at 2 and 4 months of age. This delay was adopted as a precautionary measure in the context of prior in utero belimumab exposure. No adverse events related to vaccination were reported. In the youngest child, a neuropsychiatric follow-up was performed and concluded after one year due to normal neurodevelopment.

Longitudinal maternal clinical and laboratory parameters across the three pregnancies, including SLEDAI-2K components, hematological and serological findings, urinalysis, proteinuria, blood pressure, and CLASI-A score, are summarized in Table 1.

## 3. Discussion

The management of pregnancy in women with SLE remains one of the most complex clinical challenges in rheumatology and maternal–fetal medicine, primarily due to the need to balance adequate disease control with fetal safety. Although corticosteroids, antimalarials (particularly hydroxychloroquine), and selected immunosuppressive agents (azathioprine, cyclosporine, and tacrolimus) are considered compatible with pregnancy, these therapies may be insufficient in patients with highly active disease or significant organ involvement. It is now widely recognized that maternal disease activity represents a major determinant of adverse obstetric and perinatal outcomes, including preeclampsia, preterm delivery, and fetal growth restriction. Consequently, maintaining disease remission or low disease activity is considered a cornerstone of optimal pregnancy management in SLE [10,11].

Belimumab, a humanized monoclonal antibody targeting B-lymphocyte stimulator (BLyS/BAFF), is currently approved for the treatment of both renal and extrarenal SLE [4,5]. Historically, its use during pregnancy has been discouraged due to the scarcity of safety data, despite the growing clinical need for effective therapeutic options in patients with active disease [12]. In recent years, however, evidence on belimumab exposure during pregnancy the literature has progressively accumulated, contributing to a more balanced reassessment of its risk–benefit profile.

The Belimumab Pregnancy Registry (BPR) was established to collect data on pregnancy outcomes following exposure to belimumab. Analyses published up to 2021 reported 55 prospective pregnancies with known outcomes, of which 53 resulted in live births; among these, 10 infants had major congenital anomalies. However, no specific pattern of malformations attributable to belimumab was identified, and the available data were considered insufficient to define a direct drug-related risk, given the limited number of cases and incomplete information regarding maternal disease activity and concomitant therapies [8]. A subsequent integrated analysis of 319 pregnancies exposed to belimumab, derived from clinical trials, post-marketing pharmacovigilance reports, and the BPR, demonstrated marked heterogeneity in the rates of birth defects across data sources (5.6% in clinical trials, 21.7% in the prospective BPR, and 1.1% in spontaneous reports), without the identification of a consistent teratogenic signal. Similarly, rates of pregnancy loss varied widely among reports and were difficult to interpret, as the effects of belimumab could not be disentangled from those of active SLE or concomitant treatments [7].

Additional evidence is provided by real-world observational studies. In a Taiwanese cohort including 13 pregnancies exposed to belimumab, no apparent fetal anomalies were reported, and 84.6% of pregnancies resulted in term live births [13]. Similarly, a small case series of four patients comparing women who discontinued belimumab during pregnancy with those who continued treatment beyond the early gestational weeks did not identify significant adverse maternal or fetal outcomes among term neonates, although interpretation is limited by the small sample size [14].

Published case reports further describe heterogeneous patterns of belimumab exposure, including exposure limited to the first weeks of gestation [15], initiation during pregnancy due to disease reactivation [16], or continuation throughout the entire gestational period. Overall, these reports describe generally favorable or neutral neonatal outcomes, with only rare, isolated anomalies lacking a clear causal relationship with belimumab [17]. More recently, pharmacokinetic and immunological studies have demonstrated that in pregnancies exposed to belimumab, the drug is detectable in cord blood and in neonates, confirming placental transfer; however, this exposure has not been associated with clinically significant complications during short-term follow-up [18].

Supporting this, a recent case report from the Belimumab Pregnancy Registry provided detailed longitudinal data on transplacental passage and neonatal outcomes [19]. In a mother–child pair, belimumab was detected in cord blood at birth despite the last maternal infusion occurring in the late second trimester. B-cell numbers in the neonate were reduced at birth but normalized by four months, when belimumab was no longer detectable. The child exhibited normal growth and development, received routine vaccinations, and experienced no serious infections during follow-up, providing strong evidence that in utero exposure to belimumab does not result in clinically significant short-term adverse effects on the newborn.

Taken together, the available data do not show an increased frequency of congenital malformations nor a specific pattern of anomalies in children exposed to belimumab during pregnancy, both of which are key criteria for establishing a teratogenic effect. This is consistent with the expected pharmacokinetic profile of monoclonal antibodies, which are unlikely to reach the fetus during the first trimester. Nevertheless, definitive conclusions cannot yet be drawn, and the results should be interpreted in the context of potential confounding factors.

The present case provides an original contribution to the literature by describing three consecutive pregnancies in the same patient with SLE, allowing for an intra-individual comparison of different belimumab exposure strategies over a time span (2019–2024) characterized by a progressive evolution of scientific evidence and clinical recommendations. Two distinct approaches were adopted: complete discontinuation of belimumab at conception during the first pregnancy, and continuation of treatment until gestational week 20 during the second and third pregnancies. These decisions were informed by the evidence available at the time of each pregnancy, which was extremely limited in 2019 and more robust in 2022 and 2024, particularly in light of the recommendations issued by the British Society for Rheumatology [9].

In this case, discontinuation of belimumab at conception during the first pregnancy was followed by a clinically significant disease flare in late gestation and in the postpartum period. In the second pregnancy, which occurred during a period of stable disease, continuation of belimumab until gestational week 20 was associated with good disease control during the first and second trimesters and only mild disease reactivation near term.

The third pregnancy was unplanned and occurred shortly after the flare that had developed during the third trimester of the second pregnancy. Belimumab was only continued until gestational week 20, and the patient experienced a severe disease flare in the third trimester, requiring high-dose intravenous corticosteroids. It is important to acknowledge that the observed outcomes—including disease flares and neonatal complications—cannot be attributed solely to the belimumab discontinuation strategy, as they are confounded by multiple factors, including baseline maternal disease activity, the unplanned nature of the third pregnancy, and exposure to other immunosuppressive agents, particularly corticosteroids and cyclosporine.

Indeed, the newborn developed neonatal sepsis and meningitis caused by *Streptococcus agalactiae*. This complication occurred in the context of several concurrent risk factors: high maternal disease activity during the third trimester, exposure to high-dose corticosteroids administered for the management of the lupus flare, and ongoing cyclosporine therapy. Maternal corticosteroid use during pregnancy is a well-recognized independent risk factor for neonatal infections [20], and the contribution of each individual factor cannot be disentangled in this case. Therefore, a direct causal relationship between belimumab exposure (or its discontinuation) and neonatal infection cannot be established.

Importantly, across all three pregnancies, no structural congenital anomalies, placental insufficiency, or fetal growth restriction were observed. Overall, these outcomes align with current evidence, which does not indicate a substantial increase in teratogenic risk or pregnancy loss associated with belimumab beyond the baseline risk inherent to SLE itself.

From a pharmacokinetic perspective, IgG1 monoclonal antibodies such as belimumab are expected to show minimal placental transfer during early pregnancy, with transplacental passage increasing later in gestation, especially after mid-pregnancy [21,22,23]. This transfer is mediated primarily by the neonatal Fc receptor (FcRn) expressed on placental syncytiotrophoblasts, with the largest amount of IgG transferred during the third trimester [24]. In our patient, belimumab was discontinued at gestational week 20 during the second and third pregnancies. Given the terminal half-life of belimumab of approximately 19 days, fetal exposure during late pregnancy was likely low, although it cannot be entirely excluded. This appears consistent with the absence of congenital anomalies or other evident short-term complications potentially attributable to fetal drug exposure. Conversely, treatment discontinuation at week 20 may have contributed to reduced maternal disease control, as suggested by the third-trimester flares observed in both pregnancies. These observations underscore the difficulty of balancing maternal disease stability against theoretical concerns about fetal exposure.

These pharmacokinetic considerations are also relevant when interpreting postnatal management, particularly vaccination timing. In the second and third children, rotavirus vaccination was delayed by six months compared with the standard schedule. This was a precautionary decision taken in the context of in utero belimumab exposure, rather than one based on specific recommendations for belimumab-exposed infants, for which evidence remains scarce. Given that belimumab had been discontinued at gestational week 20, fetal exposure during the phase of maximal placental transfer was likely limited. Therefore, the delayed administration of rotavirus vaccine should be interpreted as a cautious approach adopted in a setting of clinical uncertainty, rather than as a universally recommended strategy. Emerging data suggest that rotavirus vaccination may be safely administered to infants exposed to biologic agents in utero [25,26,27]. Importantly, no adverse events related to vaccination and no recurrent or severe infections beyond the neonatal period were observed during follow-up.

In this context, continuation of belimumab at least until mid-gestation—and, in selected cases, even throughout pregnancy—may be considered in women with active or unstable SLE as part of an individualized benefit–risk assessment aimed at maintaining maternal disease control and minimizing adverse outcomes related to disease activity and prolonged corticosteroid exposure.

The 2024 EULAR recommendations support this approach, stating that belimumab may be considered throughout pregnancy in women at high risk of disease reactivation, provided that the expected maternal benefits outweigh potential fetal risks. This represents a shift from earlier guidance advocating routine pre-conception discontinuation, highlighting the importance of a personalized, risk-adapted management strategy in SLE pregnancies [6].

Despite the growing body of available data, important limitations remain. Most existing evidence derives from case series or retrospective studies and is subject to confounding factors related to disease activity, concomitant therapies, and patient selection. Furthermore, pregnancy registries have not yet reached sufficient sample sizes to allow robust statistical analyses, and relevant gaps persist regarding long-term outcomes in children exposed in utero, particularly with respect to immune system development and the administration of live vaccines during early infancy.

While larger prospective studies are awaited, cases such as the present one contribute to framing belimumab use in pregnancy within a benefit–risk assessment-driven and shared decision-making approach. In this context, transparent communication of the available evidence is essential to support informed patient choices, particularly when the potential benefits of sustained maternal disease control may outweigh concerns related to an as yet unproven fetal risk.

## 4. Conclusions

In conclusion, this case provides longitudinal observations from three consecutive pregnancies in the same patient, offering a unique opportunity for intra-individual comparison of different belimumab exposure strategies over time. Although no generalizations can be drawn from a single case, and the observed outcomes are confounded by multiple factors—including baseline disease activity, the unplanned nature of one pregnancy, and concurrent immunosuppressive therapies—several considerations emerge.

First, this case underscores the importance of an individualized benefit–risk assessment in the management of pregnant patients with active SLE, particularly to minimize adverse outcomes related to uncontrolled maternal disease and prolonged corticosteroid exposure. Second, it highlights the complexity of attributing specific outcomes to any single therapeutic intervention in the context of multifactorial disease management.

Pending the availability of larger prospective studies with adequate control for confounding variables, these observations support a personalized, shared decision-making approach. In selected patients with active or difficult-to-control SLE, continuation of belimumab during pregnancy—including beyond mid-gestation—may be considered as part of an individualized benefit–risk assessment, rather than routine discontinuation at conception. Future studies should aim to disentangle the effects of biologic exposure from those of disease activity and concomitant therapies, and to provide long-term follow-up data on children exposed in utero.

## Figures and Tables

**Table 1:** Longitudinal maternal clinical and laboratory parameters across the three pregnancies and postpartum period.

	Pregnancy 1				Pregnancy 2				Pregnancy 3			
	First Trimester	Second Trimester	Third Trimester	Post- Partum	First Trimester	Second Trimester	Third Trimester	Post- Partum	First Trimester	Second Trimester	Third Trimester	Post- Partum
**Disease activity**												
Total SLEDAI-2K score	0	2	2	3	1	2	6	3	3	0	6	2
• Leukopenia	0	0	0	1	1	0	0	1	1	0	0	0
• Rash	0	2	2	2	0	2	2	2	2	0	2	2
• Arthritis	0	0	0	0	0	0	4	0	0	0	4	0
• Low complement	0	0	0	0	0	0	0	0	0	0	0	0
• Increased anti-dsDNA	0	0	0	0	0	0	0	0	0	0	0	0
CLASI-A score	0	2	19	11	0	2	2	2	2	0	4	4
**Hematology**												
Hemoglobin (g/dL)	11.4	10.7	11.2	11.5	11.3	11.5	11.5	12.9	10.7	10.3	10.7	12.2
Neutrophils/lymphocytes (/µL)	2340/440	4213/530	4420/600	690/990	1600/420	4600/700	3090/550	930/900	1020/730	2750/420	3400/500	1870/910
Platelet count (/µL)	150.000	230.000	201.000	186.000	172.000	225.000	199.000	174.000	213.000	198.000	196.000	236.000
**Biochemistry/Immunology**												
ALT/AST (U/L)	26/17	24/8	25/10	33/19	19/11	17/6	25/6	22/18	19/10	17/6	18/6	21/19
C3/C4 (mg/dL)	92/21	119/22	110/-	93/26	98/25	123/27	109/18	99/29	93/25	107/23	106/29	142/34
Anti-dsDNA titer *, IU/mL	34	20	28	35	3	15	21	21	15	14	13	1
**Renal/Urinalysis**												
Urinalysis (RBC/WBC/proteinuria)	0/0/0	0/0/0	0/0/20	3/5/0	5/10/0	0/0/0	0/5–10/0	5/5/0	0/5/0	0/5/0	5/5/0	0/0/0
24-h proteinuria (mg/24 h)	0	114	206	ND	ND	ND	ND	ND	ND	126	182	ND
**Clinical parameters**												
Blood pressure (mmHg)	100/70	105/70	110/75	120/70	95/50	100/70	120/80	116/86	120/70	110/70	100/70	145/95

ALT, alanine aminotransferase; AST, aspartate aminotransferase; RBC, red blood cell; WBC, white blood cell; CLASI-A, Cutaneous Lupus Erythematosus Disease Area and Severity Index–Activity. * Indirect immunofluorescence was performed when chemiluminescence anti-dsDNA values were >35.

## Data Availability

The data supporting the findings of this study are not publicly available due to patient privacy and ethical restrictions.

## References

[1] Petri M. (2020). Pregnancy and Systemic Lupus Erythematosus. Best Pract. Res. Clin. Obstet. Gynaecol..

[2] Wind M., Fierro J.J., Bloemenkamp K.W.M., de Leeuw K., Lely A.T., Limper M., Sueters M., Teng Y.K.O., Walter I.J., Kooiman J. (2024). Pregnancy Outcome Predictors in Systemic Lupus Erythematosus: A Systematic Review and Meta-Analysis. Lancet Rheumatol..

[3] Bremme K., Honkanen S., Gunnarsson I., Chaireti R. (2021). The Presence of Lupus Nephritis Additionally Increases the Risk of Preeclampsia among Pregnant Women with Systemic Lupus Erythematosus. Lupus.

[4] Fanouriakis A., Kostopoulou M., Andersen J., Aringer M., Arnaud L., Bae S.C., Boletis J., Bruce I.N., Cervera R., Doria A. (2023). EULAR Recommendations for the Management of Systemic Lupus Erythematosus: 2023 Update. Ann. Rheum. Dis..

[5] Fanouriakis A., Kostopoulou M., Anders H.-J., Andersen J., Aringer M., Beresford M.W., Doria A., Frangou E., Furie R., Gladman D.D. (2025). EULAR Recommendations for the Management of Systemic Lupus Erythematosus with Kidney Involvement: 2025 Update. Ann. Rheum. Dis..

[6] Rüegg L., Pluma A., Hamroun S., Cecchi I., Perez-Garcia L.F., Anderson P.O., Andreoli L., Wirström S.B., Boyadhzieva V., Chambers C. (2025). EULAR Recommendations for Use of Antirheumatic Drugs in Reproduction, Pregnancy, and Lactation: 2024 Update. Ann. Rheum. Dis..

[7] Petri M., Landy H., Clowse M.E.B., Gemzoe K., Khamashta M., Kurtinecz M., Levy R.A., Liu A., Marino R., Meizlik P. (2022). Belimumab Use during Pregnancy: A Summary of Birth Defects and Pregnancy Loss from Belimumab Clinical Trials, a Pregnancy Registry and Postmarketing Reports. Ann. Rheum. Dis..

[8] Juliao P., Wurst K., Pimenta J.M., Gemzoe K., Landy H., Moody M.A., Tilson H., Covington D., Moore T., Marino R. (2023). Belimumab Use during Pregnancy: Interim Results of the Belimumab Pregnancy Registry. Birth Defects Res..

[9] Russell M.D., Dey M., Flint J., Davie P., Allen A., Crossley A., Frishman M., Gayed M., Hodson K., Khamashta M. (2023). British Society for Rheumatology Guideline on Prescribing Drugs in Pregnancy and Breastfeeding: Immunomodulatory Anti-Rheumatic Drugs and Corticosteroids. Rheumatology.

[10] Giannakaki A.G., Giannakaki M.N., Bothou A., Nikolettos K., Machairiotis N., Pappa K.I., Tsikouras P. (2025). Current Approaches to the Management of Rheumatic Diseases in Pregnancy: Risk Stratification, Therapeutic Advances, and Maternal-Fetal Outcomes. J. Pers. Med..

[11] Andreoli L., Bertsias G.K., Agmon-Levin N., Brown S., Cervera R., Costedoat-Chalumeau N., Doria A., Fischer-Betz R., Forger F., Moraes-Fontes M.F. (2017). EULAR Recommendations for Women’s Health and the Management of Family Planning, Assisted Reproduction, Pregnancy and Menopause in Patients with Systemic Lupus Erythematosus and/or Antiphospholipid Syndrome. Ann. Rheum. Dis..

[12] Skorpen C.G., Hoeltzenbein M., Tincani A., Fischer-Betz R., Elefant E., Chambers C., Da Silva J., Nelson-Piercy C., Cetin I., Costedoat-Chalumeau N. (2016). The EULAR Points to Consider for Use of Antirheumatic Drugs before Pregnancy, and during Pregnancy and Lactation. Ann. Rheum. Dis..

[13] Kao J.H., Lan T.Y., Lu C.H., Cheng C.F., Huang Y.M., Shen C.Y., Hsieh S.C. (2021). Pregnancy Outcomes in Patients Treated with Belimumab: Report from Real-World Experience. Semin. Arthritis Rheum..

[14] Lai Y., Li B., Huang J., Du J., Yue M., Shen X., Liu X., Huang L., Lin J., Yang A. (2023). Different Pregnancy Outcomes in Patients with Systemic Lupus Erythematosus Treated with Belimumab. Lupus.

[15] Nakai T., Ikeda Y., Yamaguchi K., Asano T., Iwata F., Kidoguchi G., Fukui S., Ozawa H., Kawaai S., Kitada A. (2022). A Case Report of Two Systemic Lupus Erythematosus Pregnancies with Early Placental Exposure to Belimumab: Case Report with Review. Mod. Rheumatol. Case Rep..

[16] Wei S.R., Zhu Z.Z., Xu J., Mo H.Y. (2023). Favorable Pregnancy Outcomes in Two Patients with Systemic Lupus Erythematosus Treated with Belimumab. Int. J. Rheum. Dis..

[17] Danve A., Perry L., Deodhar A. (2014). Use of Belimumab throughout Pregnancy to Treat Active Systemic Lupus Erythematosus-A Case Report. Semin. Arthritis Rheum..

[18] Higuchi A., Saito J., Fujimori K., Ishikawa T., Kawasaki H., Miyagawa E., Abe S., Kohno C., Takai C., Sano Y. (2025). Placental and Breast Milk Transfer of Belimumab in Three Patients with Syst Emic Lupus Erythematosus Treated throughout Pregnancy. Mod. Rheumatol. Case Rep..

[19] Bitter H., Warren D.J., Bolstad N., Noraas A.L., Ostensen M.E. (2023). Transplacental Passage of Belimumab during Pregnancy and Follow-up of a Child Exposed in Utero. Ann. Rheum. Dis..

[20] Yao T.C., Chang S.M., Wu C.S., Tsai Y.F., Sheen K.H., Hong X., Chen H.Y., Wu A.C., Tsai H.J. (2023). Association between Antenatal Corticosteroids and Risk of Serious Infection in Children: Nationwide Cohort Study. BMJ.

[21] Clements T., Rice T.F., Vamvakas G., Barnett S., Barnes M., Donaldson B., Jones C.E., Kampmann B., Holder B. (2020). Update on Transplacental Transfer of IgG Subclasses: Impact of Maternal and Fetal Factors. Front. Immunol..

[22] Bussel J.B., Cines D.B., Blumberg R.S. (2025). Neonatal Fc Receptor—Biology and Therapeutics. N. Engl. J. Med..

[23] Palmeira P., Quinello C., Silveira-Lessa A.L., Zago C.A., Carneiro-Sampaio M. (2012). IgG Placental Transfer in Healthy and Pathological Pregnancies. Clin. Dev. Immunol..

[24] Marchant A., Sadarangani M., Garand M., Dauby N., Verhasselt V., Pereira L., Bjornson G., Jones C.E., Halperin S.A., Edwards K.M. (2017). Maternal Immunisation: Collaborating with Mother Nature. Lancet Infect. Dis..

[25] Fitzpatrick T., Alsager K., Sadarangani M., Pham-Huy A., Murguía-Favela L., Morris S.K., Seow C.H., Piché-Renaud P.P., Jadavji T., Vanderkooi O.G. (2023). Immunological Effects and Safety of Live Rotavirus Vaccination after Antenatal Exposure to Immunomodulatory Biologic Agents: A Prospective Cohort Study from the Canadian Immunization Research Network. Lancet Child Adolesc. Health.

[26] Schell T.L., Fass L., Hitchcock M.E., Farraye F.A., Hayney M.S., Saha S., Caldera F. (2025). Safety of Rotavirus Vaccination in Infants That Were Exposed to Biologics In Utero: A Systematic Review. Inflamm. Bowel Dis..

[27] Mahadevan U., Seow C.H., Barnes E.L., Chaparro M., Flanagan E., Friedman S., Julsgaard M., Kane S., Ng S., Torres J. (2025). Global Consensus Statement on the Management of Pregnancy in Inflammatory Bowel Disease. Inflamm. Bowel Dis..

